# Supplementary material for: Cryo-EM structures of higher order Gephyrin oligomers reveal principles of inhibitory postsynaptic scaffold organization
Source: Nat Commun. 2026 Apr 16;17:3541. doi: 10.1038/s41467-026-71771-8 (PMC13086950; doi:10.1038/s41467-026-71771-8)
Supplement: Supplementary file 1 — Supplementary Information [file 41467_2026_71771_MOESM1_ESM.pdf]

Cryo-EM Structures of Higher Order Gephyrin Oligomers Reveal Principles of Inhibitory Postsynaptic Scaffold Organization

Diego Ortiz-López[1], Tamsanqa T. Hove[1], Christiane Huhn[1], Serena Camuso[2], Pia M. van gen Hassend[1], Bodo Sander[1,*], Benjamin F.N. Campbell[3], Shiva K. Tyagarajan[3,**], Andreas Plückthun[4], Christian G. Specht[2], Hans M. Maric[1], Bettina Böttcher[1] and Hermann Schindelin[1]

[1] University of Würzburg, Rudolf Virchow Center for Integrative and Translational Bioimaging, Josef-Schneider-Str. 2, 97070 Würzburg, Germany

[2] Neuro-Bicêtre, Inserm U1195, Université Paris-Saclay, Bâtiment Gregory Pincus, 80 Rue du Général Leclerc, 94276 Le Kremlin-Bicêtre, France

[3] University of Zürich, Institute of Pharmacology and Toxicology, Winterthurerstr. 190, 8057 Zürich, Switzerland

[4] University of Zürich, Department of Biochemistry, Winterthurerstr. 190, 8057 Zürich, Switzerland

*Present address: CSL Innovation GmbH, Marburg, Germany

**Present address: Department of Life Sciences, Center for Neuroscience and Cell Biology, University of Coimbra, 3004-517 Coimbra, Portugal

Author to whom correspondence should be addressed:

hermann.schindelin@uni-wuerzburg.de

Supplementary Fig1: GephFL architecture and GephE subdomains.

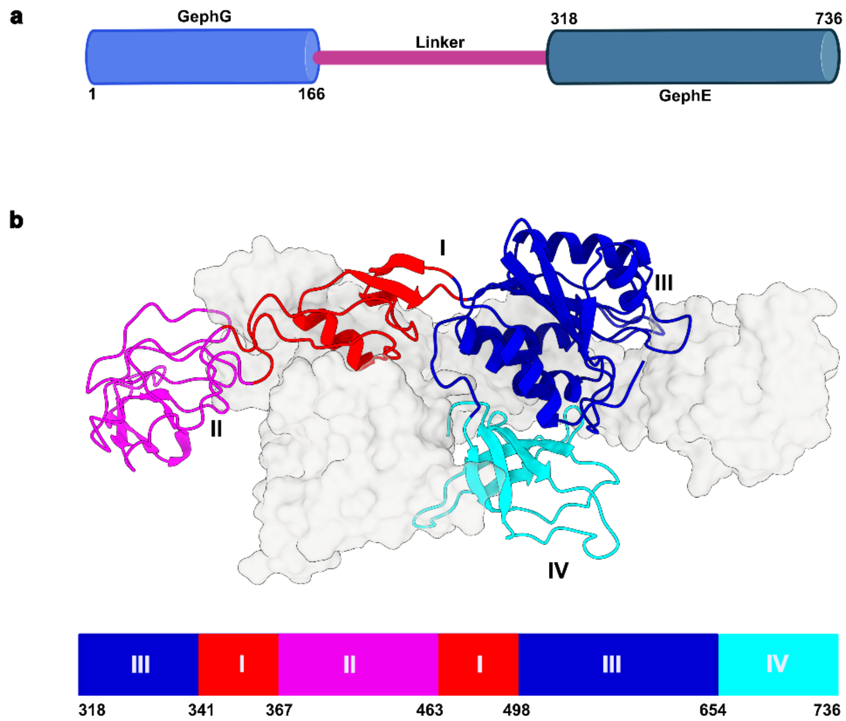

a Domain architecture of GephFL with the N-terminal G-domain (GephG), central unstructured linker and C-terminal E-domain (GephE). **b** Structure of the GephE dimer with one subunit in ribbon representation, color coded to reflect its organization into four subdomains (designated I-IV) and the other subunit in surface representation in gray. Residue numbers define the subdomain boundaries. This figure was partially created in Biorender: Ortiz-Lopez, D. https://BioRender.com/7wdnjjr

Supplementary Fig. 2: GephFL purification: Characterization of fraction A4 and SDS-PAGE analysis of selected fractions.

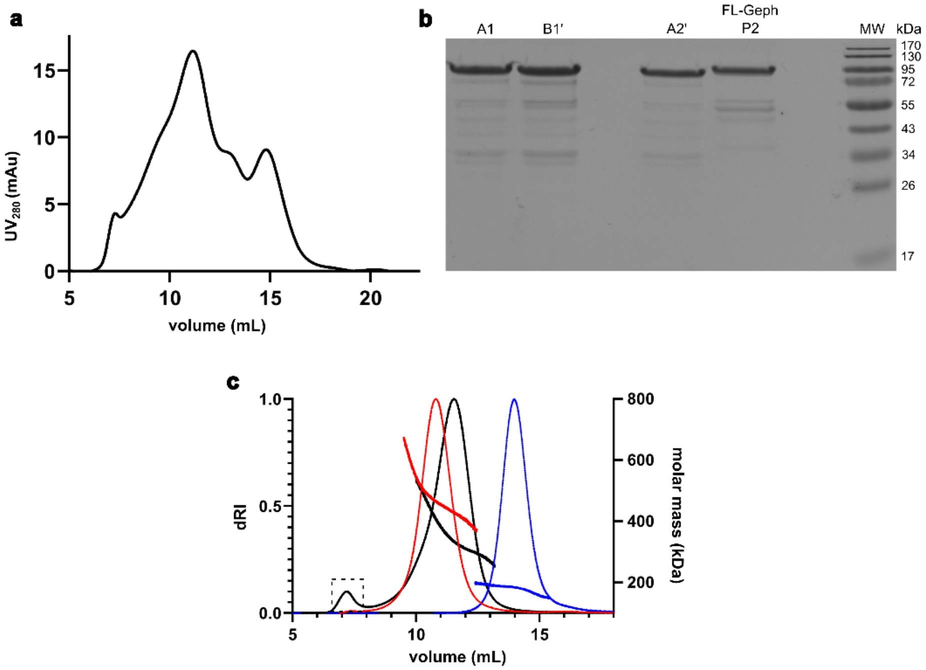

(a) Size exclusion chromatogram of GephFL fraction A4. Several peaks are present and hence this fraction was not further characterized. (b) SDS-PAGE of fractions A1, A2' and B1' of P1 GephFL (elutes at a molecular weight of 82 kDa) and GephFL P2 (elutes at a molecular weight of 84 kDa). Bands at lower molecular weights represent degradation products which are commonly observed after gephyrin purification. GephFL P2 was further analyzed in Fig. S8. (c) SEC MALS analyses of fractions A1 (red), A2' (black) and B1' (blue) across the entire chromatograms. A higher molecular weight species in A2' is highlighted by the dashed rectangle.

Supplementary Fig. 3: CryoEM data processing workflow for the GephE$_{309}$-27F3 complex.

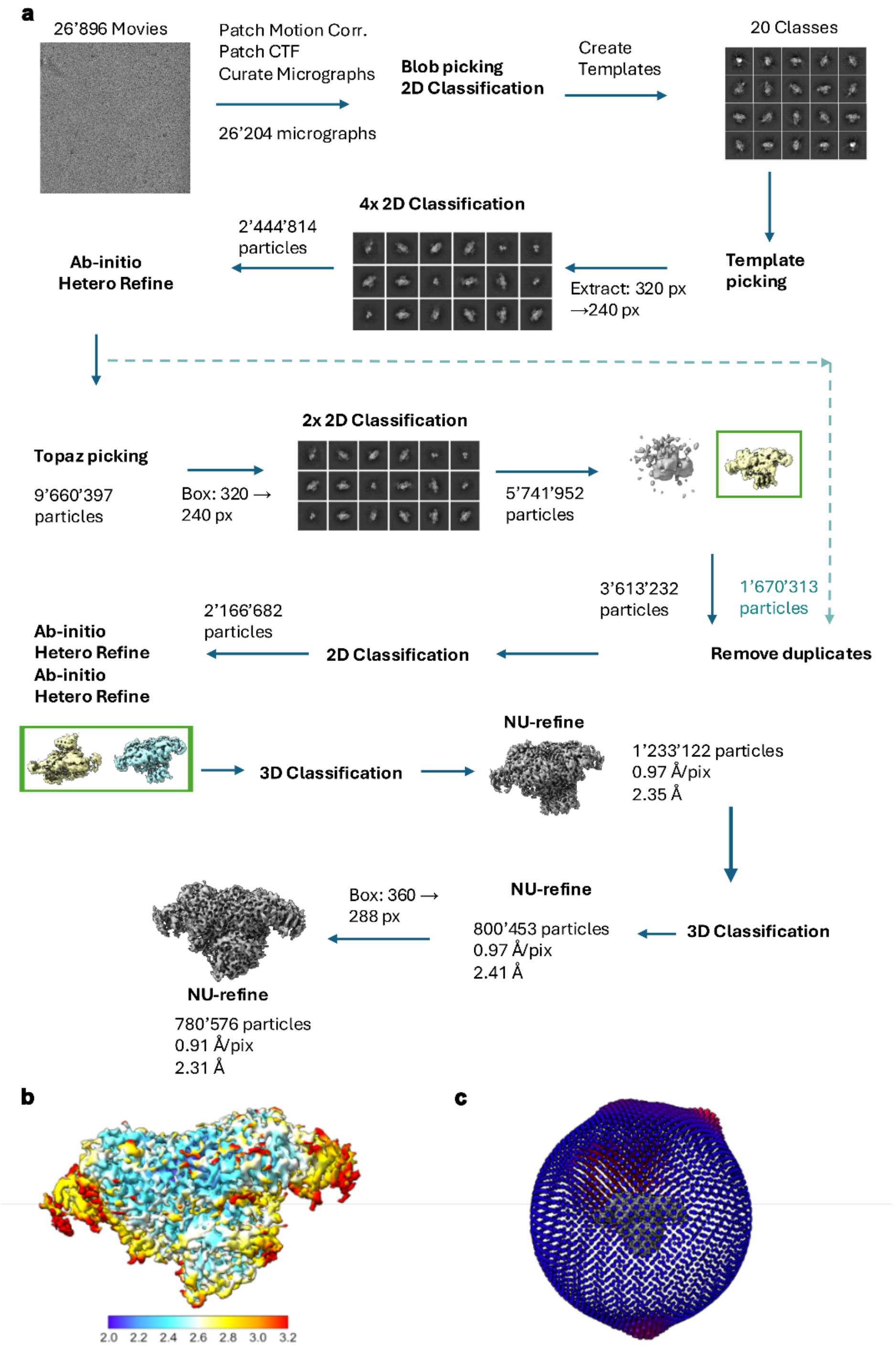

(a) CryoEM data processing from the GephE$_{309}$-273 complex, from data collection (top left), to obtaining a final 2.31 Å (b) Local resolution map from the final GephE$_{309}$-27F3 complex. (c) Angular distribution plot of the particles that contribute to the final GephE$_{309}$-27F3 map, where both heigh and color (from red to blue) are proportional to the final number of particles in that orientation.

Supplementary Fig. 4: CryoEM data processing workflow for the GephFL-27F3 complex.

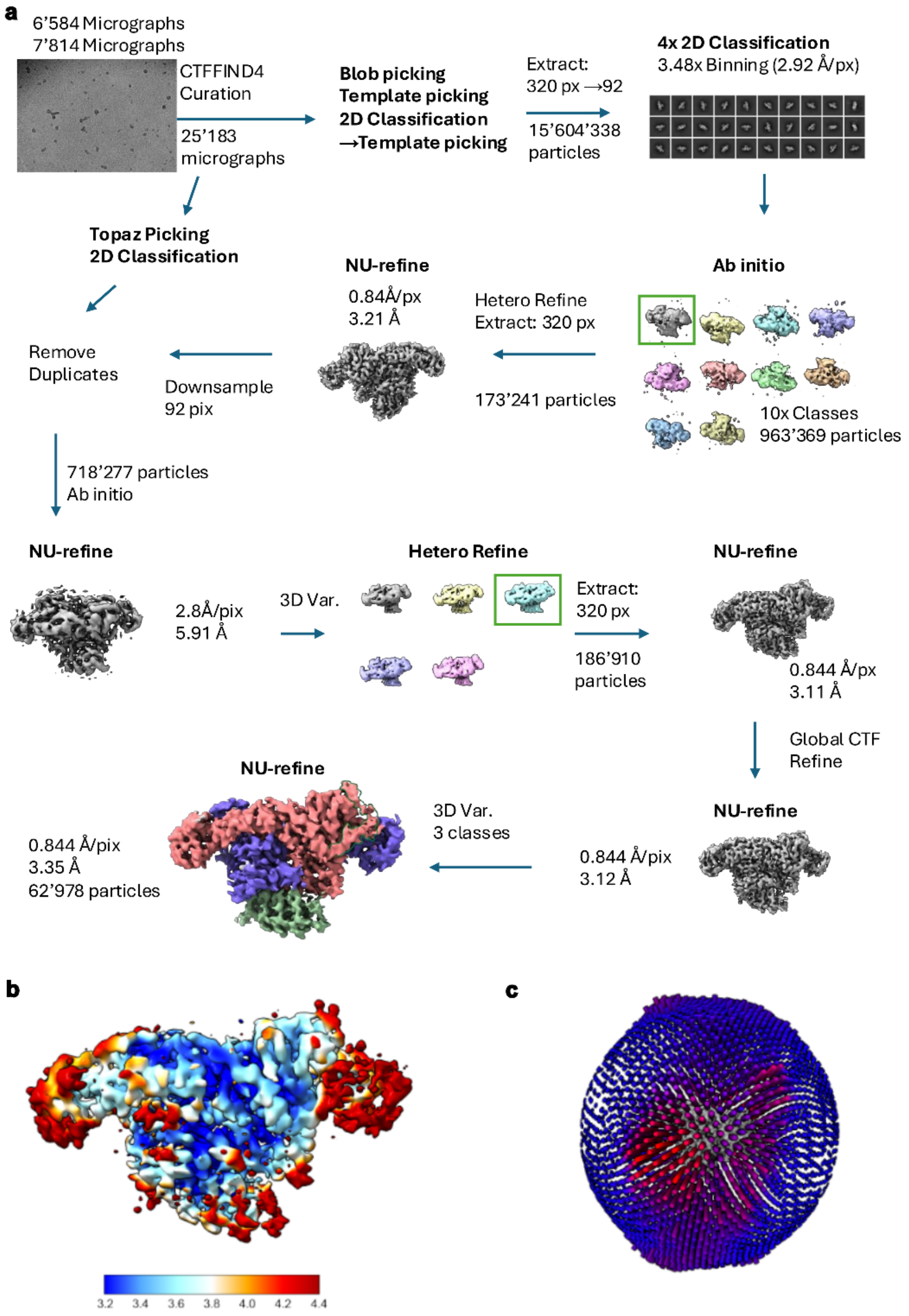

(a) CryoEM data processing from the GephFL-273 complex, from data collection (top left) to obtaining a final map at 3.35 Å resolution (b) Local resolution map from the final GephFL-27F3 complex. (c) Angular distribution plot of the particles that contribute to the final GephFL-27F3 map, where both height and color (from red to blue) are proportional to the final number of particles in that orientation.

Supplementary Fig. 5: Modelling of GlyR β-subunit binding to the GephFL-27F3 complex.

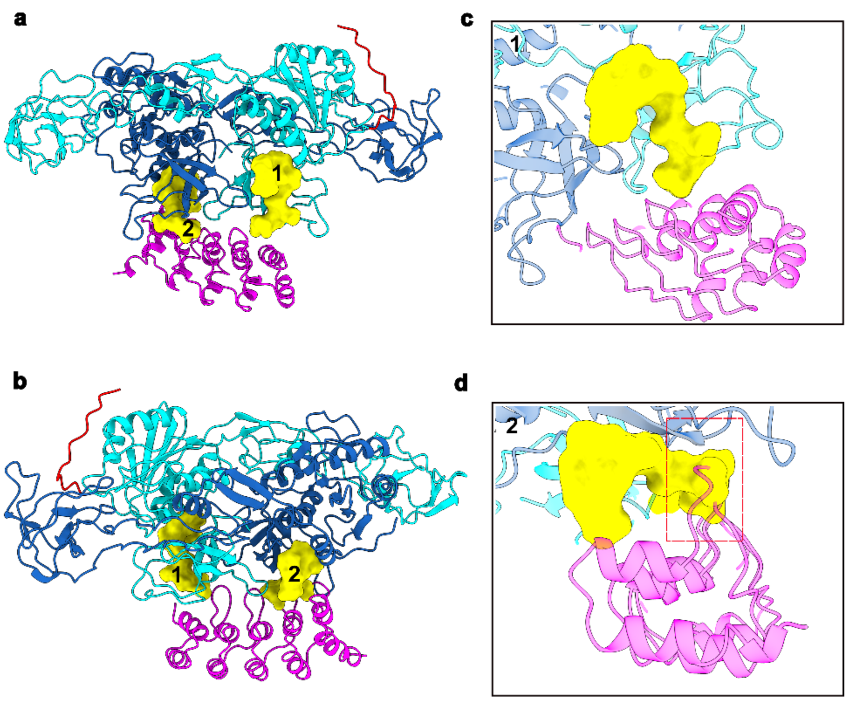

a, b Two orientations of the GephFL-27F3 complex in ribbon representation color coded as described in the main manuscript. The two GlyR β-subunit derived peptides (PDB entry 4PD1) are shown in surface representation in yellow. **c, d** Zoom into the binding sites. Binding site 1 can be occupied in the presence of 27F3 while binding of the peptide to site 2 is not compatible with 27F3 binding due to severe clashes in the region highlighted by the red rectangle.

Supplementary Fig. 6: Comparative circular dichroism spectra and SEC of GephE$_{309}$ and GephE$_{318}$.

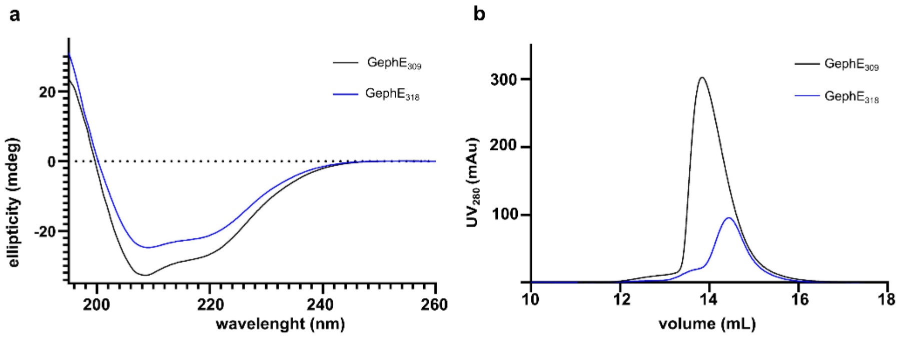

a Far UV circular dichroism (CD) spectra for GephE$_{318}$ (blue line) and GephE$_{309}$ (black line).. **b** SEC of GephE$_{318}$ (blue line) and GephE$_{309}$ (black line). SEC was performed on a Superdex 200 column. The same protocol for size exclusion chromatography was used as for GephFL, conditions and buffers are specified in the methods section.

Supplementary Fig. 7: CryoEM data processing workflow for GephFL dimer of dimers.

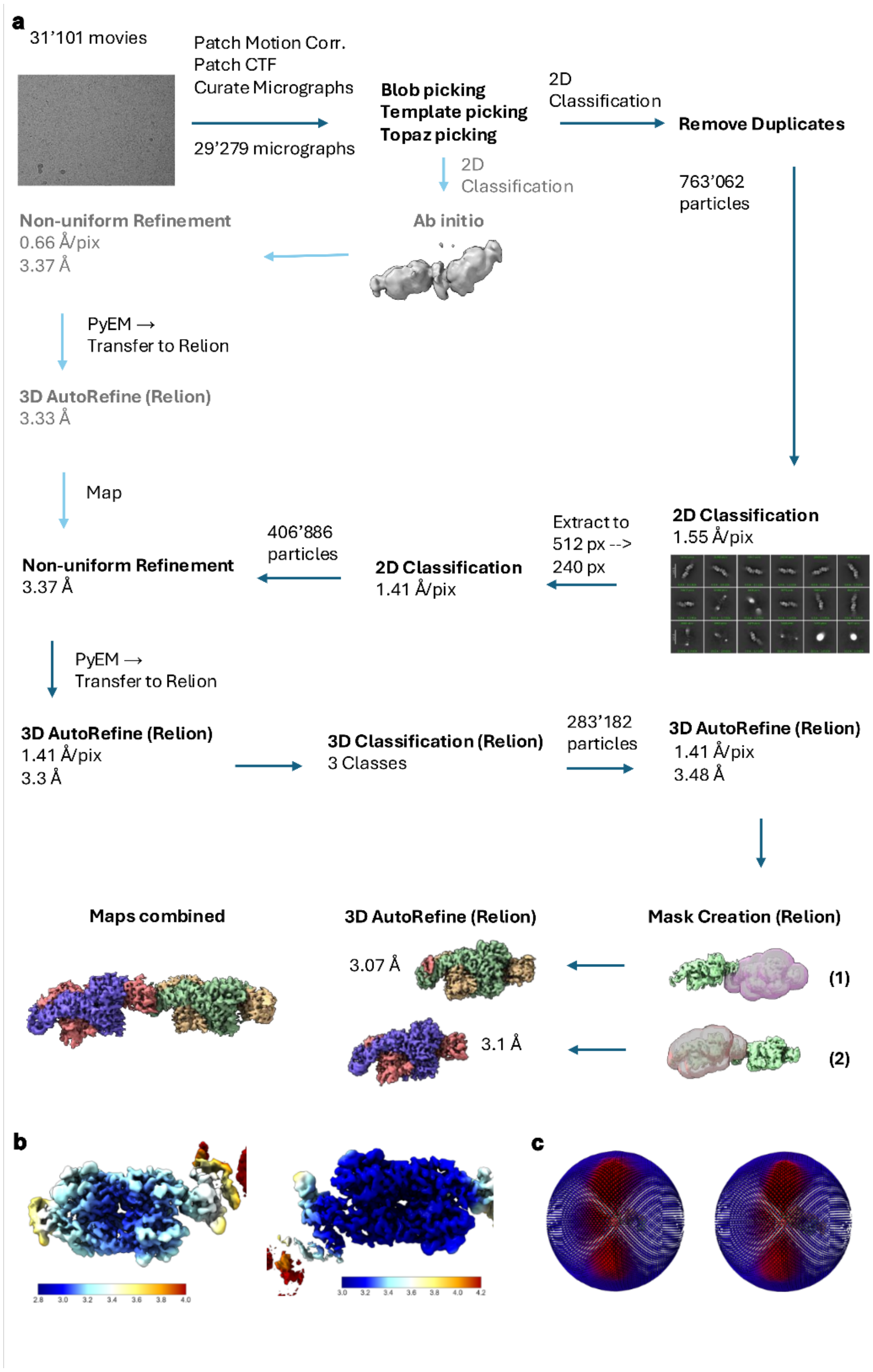

(a) CryoEM data processing from the GephFL dimer of dimers, from data collection (top left) to the local refined maps obtained independently at 3.07 Å and 3.1 Å and the final combined map. (b) Local resolution maps for the local refined maps. (c) Angular distribution plots of the particles that contribute to the final maps, where both heigh and color (from red to blue) are proportional to the final number of particles in that orientation.

Supplementary Fig. 8: Condensate formation of gephyrin wild-type and variants in HEK293T cells.

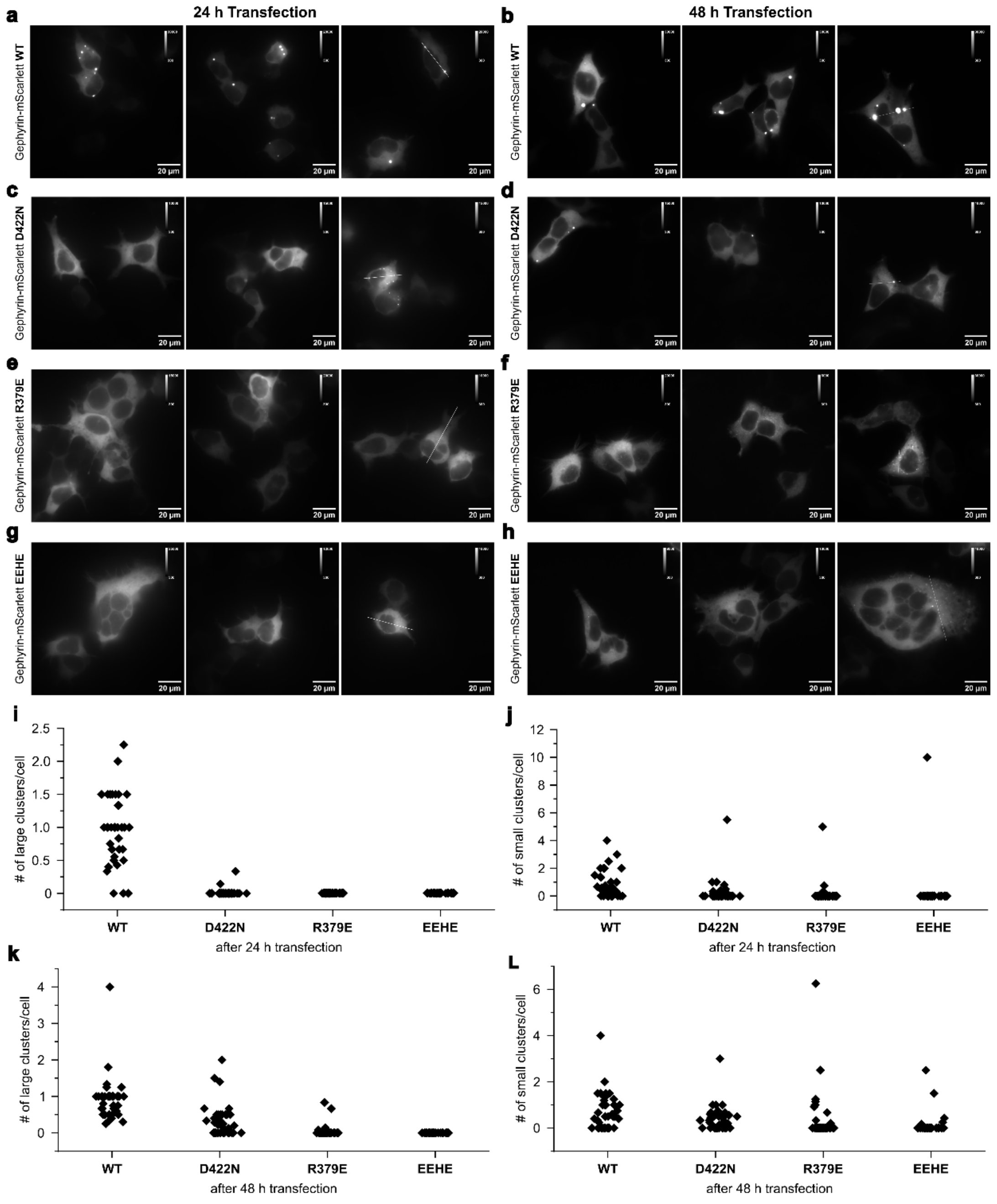

a-h Representative wide-field fluorescence microscopy images of fixed HEK293T cells overexpressing mScarlet-labeled gephyrin WT (**a**, **b**), D422N (**c**, **d**), R379E (**e**, **f**) and ^{314}EEHE^{317} (**g**, **h**) variants. Cells were transfected for 24 h (**a**, **c**, **e**, **g)** or 48 h (**b**, **d**, **f**, **h**) prior to fixation. **i-j** Cluster counts per cell (24 h transfection prior to fixation). Clusters with intensity values 5000-25000 were identified as small clusters (left), ≥25000 as large clusters (right), n≥40. **k-l** Cluster counts per cell (48 h transfection prior to fixation). Clusters with intensity values 20000-50000 were identified as small clusters (left), ≥50000 as large clusters (right), n≥40.

Supplementary Fig. 9: SEC of the GephFL dimer of dimers in the absence and presence of GlyR-βGCN4

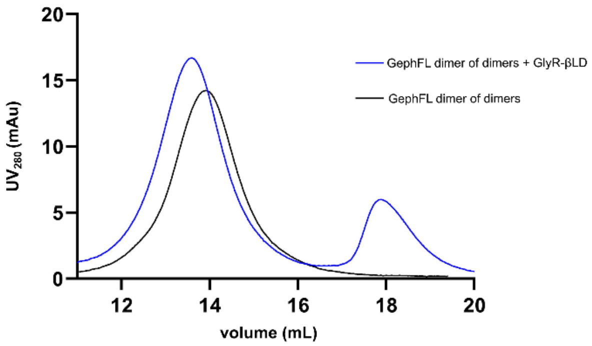

Size exclusion chromatography of the GephFL dimer of dimers in the absence (black) and presence of GlyR-βGCN4 (blue). The peak at 17.8 ml represents free GlyR-βGCN4 as this compound is present in a stoichiometric excess. Samples were analyzed on a Superose 6 column in 250 mM NaCl to avoid phase separation.

Supplementary Fig. 10: Chromatography of the GephFL P2 variant.

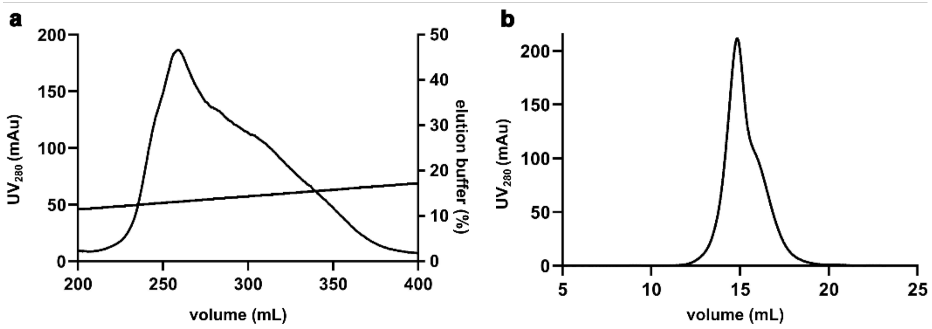

a Anion exchange chromatogram of the P2 variant. In contrast to the chromatogram of the P1 variant with two peaks and a trailing shoulder (Fig. 1b), a main peak with a broad shoulder (representing degradation products) is observed. **b** Size-exclusion chromatogram of the P2 variant using the main peak of (**a**) on a Superose 6 column. A main peak with a trailing shoulder is observed instead of the multiple oligomeric species obtained with the P1 variant (Fig. 1c). Size exclusion conditions and buffers are specified in material and methods section.

Supplementary Table 1: Plasmids and constructs.

Construct name	Encoded protein	Residues	Tags	Vector	Source	Experimental Use
$GephE_{318}$	Geph E-domain	318-736	Chitin-binding, intein fused.	pTWIN2	Ref. [1]	DSC, CD spectroscopy
$GephE_{309}$	Geph E-domain	309-736	N-terminal His_6	pET-28b	This study	Cryo-EM, DSC, CD spectroscopy
GephFL (P1)	Full-length gephyrin	1-736 (P1 splice variant)	N-terminal His_6	pET-28a	Ref. [2]	Cryo-EM, SEC-MALS
DARPin 27F3	DARPin 27F3	-	N-terminal His_6	pET-28a	Ref. [2]	Cryo-EM, binding assays
GephFL mScarlet	Full-length gephyrin	1-736 (WT)	C-terminal mScarlet	pLVX	Ref. [3]	Cellular clustering assays (HEK293, neurons), lentivirus production
GephFL mScarlet R379E	Full-length gephyrin R379E mutant	1-736	C-terminal mScarlet	pLVX	This study	Cellular clustering assays, lentivirus production
GephFL mScarlet D422N	Full-length gephyrin D422N mutant	1-736	C-terminal mScarlet	pLVX	This study	Cellular clustering assays, lentivirus production
GephFL mScarlet ^{314}EEHE^{317}	Full-length gephyrin (RRHR→EEHE)	1-736	C-terminal mScarlet	pLVX	This study	Cellular clustering assays, lentivirus production
GephFL (P2)	Full-length Gephyrin	1-750 (P2 splice variant)	N-terminal His_6	pET-28b	Ref. [4]	Control in comparative oligomerization analysis

Supplementary Table 2: Data collection and refinement statistics.

	$GephE_{309}$-27F3	GephFL-27F3	GephFLdimer of dimers
**Data collection and processing**			
**Microscope**	Titan Krios G4	Titan Krios G4	Titan Krios
**Detector**	Falcon 4i	K3	K3
**Energy Filter**	Selectris X	Quantum LS	Quantum LS
**Accelerating voltage (kV)**	300	300	300
**Magnification($x10^3$)**	165	105	130
**Number of Movies**	26204	25183	27072
**Pixel size (Å)**	0.73	0.84	0.66
**Dose $e^-/Å^2$**	44	45	42.6
**Box lengths (Å)**	70.26, 93.99, 135.96	68.88, 94.08, 137.76	71.81, 85.89, 229.50
**Box angles (°)**	90.00, 90.00, 90.00	90.00, 90.00, 90.00	90.00, 90.00, 90.00
**Resolution (Å) @FSC=0.143**	2.3	3.4	3.1
**Refinement**			
**Model resolution (Å) @FSC=0.5**	2.5	3.5	3.3
**CC mask**	0.82	0.81	0.71
**Chains**	3	3	4
**Protein residues**	967	1011	1672
**Atoms (H count)**	14784 (7422 H)	15503 (7811 H)	12749 (0 H)
**RMSD in bonds lengths (Å) / angles (°)**	0.004 Å / 0.698°	0.003 Å / 0.651°	0.004 Å / 0.692°
**Validation**			
**MolProbity score**	1.38	1.57	2.37
**Clash score**	6.22	7.24	11.85
**Rotamer outliers (%)**	0.86	0.71	6.9
**CaBLAM outliers (%)**	1.78	1.60	1.45
**Ramachandran statistics (%)**			
**Favored**	97.81	97.01	97.24
**Allowed**	2.19	2.99	2.76
**Outliers**	0.00	0.00	0.0

Supplementary Methods

Cloning and mutagenesis

Gephyrin mScarlet variants R379E, D422N, and ^{314}EEHE^{317} (R314E, R315E, R317E) were generated by site-directed mutagenesis PCR from the construct GephFL-mScarlet, encoding the Gephyrin P1 splice variant fused to mScarlet [1]. The gene encoding GephE$_{309}$ encompassing residues 309-736 was amplified by PCR and cloned using ligation into the pET-28b plasmid using the NheI and HindIII restriction enzymes sites. A list of all constructs used in this work is presented in Supplementary Table 1.

CryoEM data processing for the GephE$_{309}$-27F3 complex and structure solution

Template picked particles were extracted to a box size of 320 x 320 and cropped to 240 x 240 pixels (pixel size of 2.92 Å/pix) and screened by 2D classification in CryoSPARC [2]. After ab-initio reconstruction and sequential heterogenous refinement, 1,670,313 particles from the best classes were combined with 3,613,232 particles from Topaz picking and duplicates were removed. The joined particles were classified again using 2D classification and 2,166,682 particles from selected classes were used for an ab-initio reconstruction with 4 classes. The particles were subjected to heterogeneous refinement and the best class with 2,166,682 particles was used for a subsequent 3D classification. The best class with 1,233,122 particles was subjected to non-uniformity (NU) refinement to produce a volume with a resolution of 2.4 Å at a pixel size of 0.97 Å/pix. 3D classification with 2 classes led to a final class with 1,233,122 particles that was NU-refined to a resolution of 2.4 Å. Extraction of the particles to a box size of 288 x 288 pixels and further NU-refinement resulted in a map with 780,576 particles and a resolution of 2.31 Å. All steps are summarized in Supplementary Fig. 3.

CryoEM data processing for the GephFL-27F3 complex

Template picked particles were extracted to a box size of 320 x 320 and cropped to 92 x 92 pixels (pixel size of 2.92 Å/pix) and screened by 2D classification in CryoSPARC [2]. The resulting 963,369 particles were used for ab-initio reconstruction with 10 classes. After heterogenous refinement, particles from the best class were extracted at a box size of 320 x 320 pixels and a pixel size of 0.84 Å/pix and refined using NU refinement resulting in a resolution of 3.2 Å. Particles obtained from Topaz picking and subsequent 2D classification were joined with the refined particles and down sampled to a box size of 92 x 92 pix (2.8 Å/pix) and duplicates were removed. 718,277 particles were NU-refined and 3D variability analysis was used to generate 5 clusters. The 5 clusters were subjected to heterogenous refinement and the best class with 186,910 particles was selected. In this cluster, the presence of extra-densities in comparison with the rest of the clusters was evident. The particles from this class were extracted to a box size of 320 x 320 pixels at a pixel size of 0.84 Å/pix and were NU refined, producing a resolution of 3.1 Å. After global CTF refinement and NU-refinement, the 3D variability job was used to create 3 clusters

and the final map consisting of 62,978 particles had a resolution of 3.35 Å. All steps are summarized in Supplementary Fig. 4.

CryoEM data processing for the GephFL dimer of dimers structure

For the dimer of dimers map, multiple picking strategies were employed including blob picking, template picking and Topaz picking [2, 3]. Particles were extracted to a box size of 256 x 256 at a pixel size of 1.55 Å/pix and then subjected to several rounds of 2D classification for each picking strategy. Classes were selected based on well centered particles that represented linear dimer of dimers, leaving out hexamers and dimers. Selected particles from each picking strategy were combined and duplicates were removed. 763,062 particles remained and were extracted to a box size of 240 x 240 pixels at a pixel size of 1.41 Å/pix. After another round of 2D classification 406,886 particles remained and were refined using non-uniform refine in Cryosparc, producing a map with a nominal resolution of 3.3 Å. Using PyEM [4] the data were transferred to star format for use in RELION [5]. 3D Auto-Refine in RELION with BLUSH [6] led to a 3.3 Å map. Unaligned 3D classification produced 3 classes of which the best resolved class with 283,182 particles was chosen and further refined to a map of 3.5 Å. Local masks surrounding each dimer domain were created (Supplementary Fig. 7) and each domain was locally refined resulting in maps of 3.07 Å for one dimer and 3.1 Å for the other dimer.

Supplementary References

1. Schrader, N., et al., *Biochemical characterization of the high affinity binding between the glycine receptor and gephyrin*. J Biol Chem, 2004. **279**(18): p. 18733-41.
2. Campbell, B.F.N., et al., *A DARPin-based molecular toolset to probe gephyrin and inhibitory synapse biology*. Elife, 2022. **11**.
3. Dos Reis, R., et al., *Complex regulation of Gephyrin splicing is a determinant of inhibitory postsynaptic diversity*. Nat Commun, 2022. **13**(1): p. 3507.
4. Sander, B., et al., *Structural characterization of gephyrin by AFM and SAXS reveals a mixture of compact and extended states*. Acta Crystallogr D Biol Crystallogr, 2013. **69**(Pt 10): p. 2050-60.
